# Climate change: Nursing leadership in disaster situations

**DOI:** 10.1590/1518-8345.0000.4569

**Published:** 2025-06-02

**Authors:** Eunice da Conceição Gatinho Pires, Maria Adriana Pereira Henriques, Paulo Jorge Nogueira, Miguel André Telo de Arriaga, Andreia Cátia Jorge Silva Costa

**Affiliations:** 1 Escola Superior de Enfermagem de Lisboa, Centro de Investigação, Inovação e Desenvolvimento em Enfermagem de Lisboa, Lisboa, Portugal.; 2 Universidade de Lisboa, Faculdade de Medicina, Instituto de Saúde Ambiental, Lisboa, Portugal.; 3 Hospital do Divino Espírito Santo de Ponta Delgada, Ponta Delgada, Portugal.; 4 Universidade de Lisboa, Laboratório Associado TERRA, Lisboa, Portugal.

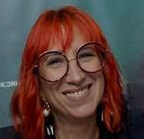

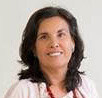

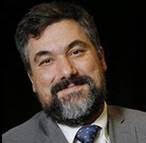

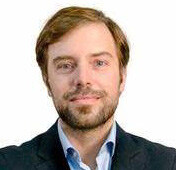

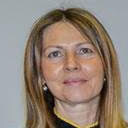











Climatic conditions are challenging, especially when combined with other factors, such as geographical conditions.

Nurses play a vital role in disaster preparedness and response, directly influencing their organization and effectiveness. However, scientific evidence still struggles to define clearly the specific knowledge and skills nurses should possess when leading in disaster situations. The central theme of this editorial aims to contribute to reflection on the recognition of the competencies of nurses who work in these situations.

To analyze this topic, we focused on a particular case of an island in the Azores archipelago. Data was collected through a documentary analysis of the Azores Regional Civil Protection Emergency Plan, the Ponta Delgada Municipal Civil Protection Emergency Plan and the Hospital External Emergency Plan, seeking to obtain in-depth knowledge of the available information.

In the documentary analysis of the government plans on the theme of leadership, the following sub-themes were identified:

**1. Coordination of Health Actions:** The lead nurse must coordinate and guarantee the efficient triage, care and evacuation of victims, ensuring that all actions are carried out in an organized and effective manner.

**2. Management of Shelter Areas:** The nurse is responsible for coordinating the shelter areas’ management, food and basic care for displaced patients, ensuring their needs are adequately met during the crisis.

**3. Organization of Field Hospitals:** The lead nurse collaborates effectively in the organization, installation and management of field hospitals or medical outposts, ensuring that the necessary infrastructure responds effectively to healthcare needs.

These sub-themes highlight the relevance of nursing leadership in disaster response, demonstrating the complexity and importance of their roles in these events.

The discussion on the importance of nurses’ leadership reveals a consensus around the relevance of this capacity for effective disaster response. Some arguments support the idea that strong leadership is fundamental to increasing nurses’ confidence during crises, allowing them to act more effectively, and that, in the “chaos” characteristic of disaster situations, the presence of a clear voice of command is crucial^(1)^. Furthermore, the need for well-prepared leaders to guide the team and promote an environment of collaboration, essential for the functioning of relief operations, is emphasized, in addition to the implementation of simplified protocols and guidelines that can support nurse leaders in emerging decision-making, ensuring greater clarity in the actions to be carried out^(2)^. The need for protocols is also reinforced by other researchers^(3)^, who claim that nurses must be integrated into health policies and risk management strategies, positioning them as central figures in the disaster response structure.

Studies point to the importance of nurses’ adaptability by arguing that professionals often expand their roles during disasters, acting as leaders, educators and decision-makers^(1)^. This ability to adapt remains an essential element, given that effective communication and collaboration between the various disciplines and organizations are key to successful crisis management^(4)^. To ensure this communication, nurse leaders must establish a continuous dialogue and also actively participate in developing new protocols and updating existing ones.

In short, nursing leadership in disaster is not just a responsibility, but an integral part of the professional role that improves the efficiency in disaster response and also contributes to the quality of care for the people involved. When empowered with knowledge, experience and training, nurses bring together communication skills between different disciplines and decision-making capacity in crisis, contributing to positive results in the care of the population.
