# Supplementary material for: Climate change: Nursing leadership in disaster situations
Source: Rev Lat Am Enfermagem. 2025 Jun 2;33:e4569. doi: 10.1590/1518-8345.0000.4569 (PMC12129446; doi:10.1590/1518-8345.0000.4569)
Supplement: Translation

# Translation: Alterações climáticas: liderança em Enfermagem em situações de catástrofe

**DOI:** 10.1590/1518-8345.0000.4570

**Published:** 2025-05-14

**Authors:** Eunice da Conceição Gatinho Pires, Maria Adriana Pereira Henriques, Paulo Jorge Nogueira, Miguel André Telo de Arriaga, Andreia Cátia Jorge Silva Costa

**Affiliations:** 1 Escola Superior de Enfermagem de Lisboa, Centro de Investigação, Inovação e Desenvolvimento em Enfermagem de Lisboa, Lisboa, Portugal.; 2 Universidade de Lisboa, Faculdade de Medicina, Instituto de Saúde Ambiental, Lisboa, Portugal.; 3 Hospital do Divino Espírito Santo de Ponta Delgada, Ponta Delgada, Portugal.; 4 Universidade de Lisboa, Laboratório Associado TERRA, Lisboa, Portugal.











As condições climáticas são desafiadoras, sobretudo associadas a outros fatores, a exemplo das condições geográficas.

Os enfermeiros desempenham um papel vital na preparação e resposta, influenciando diretamente a organização e eficácia da resposta em situações de catástrofe. As evidências científicas, entretanto, ainda enfrentam dificuldades para definir claramente os conhecimentos e competências específicas que os enfermeiros devem possuir na liderança em situações de catástrofe. O tema central deste editorial pretende contribuir para a reflexão relativa ao reconhecimento das competências dos enfermeiros que atuam nessas situações.

Na análise deste tema, dedicámo-nos a um caso particular de uma Ilha do Arquipélago dos Açores, usando como recurso a análise de textos documentais. A coleta de dados foi realizada mediante análise documental do Plano Regional de Emergência de Proteção Civil dos Açores, do Plano Municipal de Emergência de Proteção Civil de Ponta Delgada e do Plano Hospitalar de Emergência Externa buscando obter conhecimento aprofundado das informações disponíveis.

Na análise documental dos planos de governo sobre o tema da liderança, foram identificados os seguintes subtemas:

**1. Coordenação das Ações de Saúde:** O enfermeiro líder deve coordenar e garantir a triagem, o cuidado e a evacuação eficiente das vítimas, assegurando que todas as ações sejam executadas de forma organizada e eficaz.

**2. Gestão de Zonas de Abrigo:** O enfermeiro é responsável por coordenar a gestão das áreas de abrigo, alimentação e os cuidados básicos para os doentes deslocados, garantindo que as suas necessidades sejam adequadamente satisfeitas durante a crise.

**3. Organização de Hospitais de Campanha:** O enfermeiro líder colabora efetivamente na organização, instalação e gestão de hospitais de campanha ou postos médicos avançados, assegurando que a infraestrutura necessária responda de forma eficaz às necessidades dos cuidados de saúde.

Esses subtemas ressaltam a relevância do papel de liderança dos enfermeiros na resposta a catástrofes, demonstrando a complexidade e a importância de suas funções nestes eventos.

A discussão entre os autores sobre a importância da liderança dos enfermeiros revela um consenso em torno da relevância dessa capacidade para a eficácia da resposta a catástrofes. Há argumentos que sustentam que uma liderança forte é fundamental para aumentar a confiança dos enfermeiros durante as crises, permitindo que eles atuem com mais eficácia e que, no meio do “caos” característico das situações de catástrofe, a presença de uma voz de comando clara é crucial^(1)^. Ainda, é enfatizada a necessidade de líderes bem preparados para orientar a equipe e promover um ambiente de colaboração, essencial para o funcionamento das operações de socorro e que a implementação de protocolos e diretrizes simplificadas pode apoiar os enfermeiros líderes na tomada de decisão emergente, garantindo maior clareza nas ações a serem realizadas^(2)^. A necessidade de protocolos é reforçada por outros pesquisadores^(3)^, quando destacam que os enfermeiros devem estar integrados nas políticas de saúde e nas estratégias de gestão de risco, posicionando-os como figuras centrais na estrutura de resposta a catástrofes.

Os estudos também apontam para a importância da adaptabilidade dos enfermeiros ao argumentarem que os profissionais frequentemente expandem os seus papéis durante as catástrofes, atuando como líderes, educadores e tomadores de decisão^(1)^. Essa capacidade de adaptação continua sendo um elemento essencial, ao se observar que a comunicação eficaz e a colaboração entre as diversas disciplinas e organizações são determinantes para o sucesso na gestão de crises^(4)^. Para garantir essa comunicação, os enfermeiros líderes devem não apenas instaurar um diálogo contínuo, mas também participar ativamente no desenvolvimento de novos protocolos e na atualização dos já existentes.

Em suma, a liderança em Enfermagem na catástrofe não é apenas uma responsabilidade, mas uma parte integrante do papel profissional que melhora não apenas a eficiência na resposta a catástrofes, mas também contribui para a qualidade do cuidado das pessoas envolvidas. Os enfermeiros, quando capacitados com conhecimento, experiência e formação, reúnem competências de comunicação entre as diferentes disciplinas e capacidade de decisão em situações de crise, contribuindo com resultados positivos nos cuidados à população.

# Translation: Alteraciones climáticas: liderazgo en Enfermería en situaciones de catástrofe

**DOI:** 10.1590/1518-8345.0000.4568

**Published:** 2025-05-14

**Authors:** Eunice da Conceição Gatinho Pires, Maria Adriana Pereira Henriques, Paulo Jorge Nogueira, Miguel André Telo de Arriaga, Andreia Cátia Jorge Silva Costa

**Affiliations:** 1 Escola Superior de Enfermagem de Lisboa, Centro de Investigação, Inovação e Desenvolvimento em Enfermagem de Lisboa, Lisboa, Portugal.; 2 Universidade de Lisboa, Faculdade de Medicina, Instituto de Saúde Ambiental, Lisboa, Portugal.; 3 Hospital do Divino Espírito Santo de Ponta Delgada, Ponta Delgada, Portugal.; 4 Universidade de Lisboa, Laboratório Associado TERRA, Lisboa, Portugal.











Las condiciones climáticas son desafiadoras, sobre todo asociadas a otros factores, a ejemplo de las condiciones geográficas.

Los enfermeros desempeñan un papel vital en la preparación y respuesta, influyendo directamente en la organización y eficacia de la respuesta en situaciones de catástrofe. Las evidencias científicas, sin embargo, aún enfrentan dificultades para definir claramente los conocimientos y competencias específicas que los enfermeros deben poseer en el liderazgo en situaciones de catástrofe. El tema central de este editorial pretende contribuir para la reflexión relativa al reconocimiento de las competencias de los enfermeros que actúan en estas situaciones.

En el análisis de este tema, nos dedicamos a un caso particular de una isla del archipiélago de los Azores, utilizando como recurso el análisis de textos documentales. La recopilación de datos se realizó mediante análisis documental del Plan Regional de Emergencia de Protección Civil de los Azores, del Plan Municipal de Emergencia de Protección Civil de Ponta Delgada y del Plan Hospitalario de Emergencia Externa buscando obtener conocimiento profundizado de las informaciones disponibles.

En el análisis documental de los planes de gobierno sobre el tema del liderazgo, se identificaron los siguientes subtemas:

**1. Coordinación de las Acciones de Salud:** El enfermero líder debe coordinar y garantizar el triaje, el cuidado y la evacuación eficiente de las víctimas, asegurando que todas las acciones se ejecuten de manera organizada y eficaz.

**2. Gestión de Zonas de Refugio:** El enfermero es responsable por coordinar la gestión de las áreas de refugio, alimentación y los cuidados básicos para los enfermos desplazados, garantizando que sus necesidades sean adecuadamente satisfechas durante la crisis.

**3. Organización de Hospitales de Campaña:** El enfermero líder colabora efectivamente en la organización, instalación y gestión de hospitales de campaña o puestos médicos avanzados, asegurando que la infraestructura necesaria responda de manera eficaz a las necesidades de atención médica.

Estos subtemas destacan la relevancia del papel de liderazgo de los enfermeros en la respuesta a catástrofes, demostrando la complejidad y la importancia de sus funciones en estos eventos.

La discusión entre los autores sobre la importancia del liderazgo de los enfermeros revela un consenso alrededor de la relevancia de esta capacidad para la eficacia de la respuesta a catástrofes. Hay argumentos que sostienen que un liderazgo fuerte es fundamental para aumentar la confianza de los enfermeros durante las crisis, permitiendo que ellos actúen con más eficacia y que, en medio del “caos” característico de las situaciones de catástrofe, la presencia de una voz de mando clara es crucial^(1)^. Además, Kerfoot enfatiza la necesidad de líderes bien preparados para guiar al equipo y promover un ambiente de colaboración, esencial para el funcionamiento de las operaciones de socorro, y que la implementación de protocolos y directrices simplificadas puede apoyar a los enfermeros líderes en la toma de decisiones emergentes, garantizando mayor claridad en las acciones a realizar^(2)^. La necesidad de protocolos es reforzada por otros investigadores^(3)^, cuando señalan que los enfermeros deben estar integrados en las políticas de salud y en las estrategias de gestión de riesgo, posicionándolos como figuras centrales en la estructura de respuesta a catástrofes.

Los estudios también apuntan para la importancia de la adaptabilidad de los enfermeros al argumentar que los profesionales a menudo expanden sus roles durante las catástrofes, actuando como líderes, educadores y tomadores de decisión^(1)^. Esta capacidad de adaptación sigue siendo un elemento esencial, al observar que la comunicación eficaz y la colaboración entre las diversas disciplinas y organizaciones son determinantes para el éxito en la gestión de crisis^(4)^. Para garantizar esta comunicación, los enfermeros líderes deben no solo instaurar un diálogo continuo, sino también participar activamente en el desarrollo de nuevos protocolos y en la actualización de los ya existentes.

En resumen, el liderazgo en Enfermería en situaciones de catástrofe no es solo una responsabilidad, sino una parte integral del rol profesional que mejora no solo la eficiencia en la respuesta a catástrofes, sino también contribuye a la calidad del cuidado de las personas involucradas. Los enfermeros, cuando están capacitados con conocimientos, experiencia y formación, reúnen competencias de comunicación entre las diferentes disciplinas y capacidad de decisión en situaciones de crisis, contribuyendo con resultados positivos en la atención a la población.
